# Prenatal Phenotypic Features of Five Fetal Cases With *RNU4ATAC*‐Associated Microcephalic Osteodysplastic Primordial Dwarfism Type I

**DOI:** 10.1002/pd.70240

**Published:** 2026-08-14

**Authors:** Alexandra Liebmann, Tanja Richter, Nevena Krstić, Markus Hoopmann, Karl Oliver Kagan, Lena‐Sophie Menig‐Benzig, Simone Olivieri, Olaf Riess, Andreas Dufke

**Affiliations:** ^1^ Institute of Medical Genetics and Applied Genomics University Hospital Tübingen Tübingen Germany; ^2^ Center for Rare Disease University of Tübingen Tübingen Germany; ^3^ MVZ Genetikum GmbH Center for Human Genetics Neu‐Ulm Germany; ^4^ Morsani College of Medicine University of South Florida Tampa Florida USA; ^5^ Department of Women's Health University of Tübingen Tübingen Germany; ^6^ MVZ Genetikum GmbH Center for Human Genetics Stuttgart Germany

**Keywords:** fetal malformations, MOPD1, non‐coding genes, prenatal diagnosis, *RNU4ATAC*

## Abstract

**Objective:**

To present the prenatal sonographic features, genomic findings, and pregnancy outcomes of fetuses with biallelic pathogenic *RNU4ATAC* variants linked to microcephalic osteodysplastic primordial dwarfism type I (MOPD1).

**Methods:**

This retrospective case series includes five prenatal cases with MOPD1. Diagnoses were established by prenatal ultrasound and genetic testing. Genome sequencing (GS) or targeted exome sequencing (ES) detected the variants either prenatally or after termination of pregnancy (TOP). Clinical data including parental demographics, ultrasound findings, and pregnancy outcomes were collected.

**Results:**

All fetuses presented with consistent anomalies on ultrasound including intrauterine growth restriction (IUGR), microcephaly, agenesis of the corpus callosum (ACC), intracranial cysts, lissencephaly, and micrognathia. IUGR was the earliest anomaly detected in all five cases. Prenatal ultrasound findings suggestive of skeletal dysplasia were identified in one case. All cases carried biallelic pathogenic *RNU4ATAC* variants associated with MOPD1. TOP was chosen in four cases. One fetus was delivered at 39 + 1 weeks with genetic diagnosis confirmed at 27 weeks.

**Conclusion:**

IUGR, microcephaly and ACC can be detected in fetuses with MOPD1 at around 18 weeks of gestation. Interestingly, skeletal dysplasia was not a consistent prenatal finding. Variants in the non‐coding *RNU4ATAC* gene need to be detected by GS or targeted approaches beyond standard ES.

## Summary

- What is already known?
  ◦ The non‐coding *RNU4ATAC* gene is missed by standard exome sequencing. MOPD1 is a severe *RNU4ATAC*‐related disorder featuring skeletal dysplasia, short stature, microcephaly, and brain anomalies. Prenatal diagnosis remains challenging due to limited prenatal phenotyping data.
- What does this study add?
  ◦ IUGR at 18 weeks is the earliest identified prenatal ultrasound finding.
  ◦ Skeletal dysplasia, though defining in MOPD1, is inconsistently detected prenatally.
  ◦ Genome or targeted exome sequencing prenatally improves detection of non‐coding variants.

## 1 Introduction

Prenatal diagnosis of genetic syndromes with structural anomalies remains challenging, particularly when phenotypic findings are variable and non‐specific. With the rise of prenatal next generation sequencing, the diagnostic yield in this setting has increased, enabling the identification of novel gene‐disease associations. The *RNU4ATAC* gene encodes U4atac small nuclear RNA, an essential component of the minor spliceosome required for the accurate splicing of U12‐type introns (MIM* 601428) [1]. Biallelic pathogenic variants in *RNU4ATAC* cause a spectrum of rare autosomal recessive disorders, collectively referred to as RNU4ATAC‐opathies, including MOPD1, Roifman syndrome, and Lowry‐Wood syndrome. The phenotypic spectrum of these disorders significantly overlaps, with MOPD1 representing the most severe and often fatal form. Postnatally, patients with MOPD1 are characterized by microcephaly, growth failure, skeletal dysplasia, and neurodevelopmental delay [2, 3]. However, prenatal manifestations of this disorder remain largely undefined [4, 5, 6].

This study describes the prenatal phenotypic profile observed in 5 fetuses harboring biallelic pathogenic *RNU4ATAC* variants located in the 5′ stem loop region, which have previously been associated with MOPD1 in postnatal cases.

## 2 Methods

We solicited investigators from the Fetal Sequencing Consortium (FSC) for prenatal MOPD1 cases [7]. In total, we collected five fetal cases from four unrelated families and non‐consanguineous couples (Table 1). All parents were non‐Hispanic white. Pregnancies were conceived naturally. Abnormal prenatal ultrasound findings prompted invasive genetic testing in all cases. Cell‐free DNA testing (NIPT) was performed in three cases. Fetal karyotyping and chromosomal microarray analysis (CMA) were performed in all 5 cases. Cases 1 and 2 were fetuses from the same couple. In both cases CVS was performed at 20 + 2 weeks. Case 1 was diagnosed retrospectively via sanger sequencing after the diagnosis was established in Case 2. In Cases 3 and 4, amniocentesis was performed at 24 + 6 weeks and 23 + 1 weeks, respectively. In Case 5, genetic testing was performed on DNA extracted from formalin‐fixed paraffin‐embedded (FFPE) placental tissue after TOP.

**TABLE 1:** Genetic investigation and clinical information of fetuses diagnosed with MOPD1.

Case	MA (yrs)	First trimester US screening	Earliest detected ultrasound anomalies	RNU4ATAC nucleotide changes *	Type of genetic sequencing (tissue; timing result)	Pregnancy outcome	Previous pregnancies
1	32	Normal at 10 + 5 weeks	IUGR at 18 + 5 weeks	NR_023343.3: n.[51G > A]; [51G > A]	Segregation (CVS; 3 years after TOP)	TOP at 22 + 0 weeks	1 miscarriage
2	35	Normal at 11 + 6 weeks	IUGR and ACC at 20 + 1 weeks	NR_023343.3: n.[51G > A]; [51G > A]	Trio genome sequencing (CVS; 30 days afterCVS/TOP)	TOP at 20 + 6 weeks	2 miscarriages 1 TOP (Case 1) 1 healthy son
3	25	N/A	IUGR at 20 + 2 weeks	NR_023343.3: n.[51G > A]; [51G > A]	Targeted trio exome sequencing (AC; 15 days after AC)	Delivered at 39 + 1 weeks; died at 30 months of age	1 miscarriage 1 healthy daughter 1 healthy son
4	24	N/A	IUGR at 23 + 1 weeks	NR_023343.3: n.[30G > A]; [55G > A]	Targeted trio exome sequencing (AC; 16 days after AC)	TOP at 27 weeks	—
5	30	Normal at 14 + 2 weeks	IUGR and echogenic focus at 18 + 2 weeks	NR_023343.3: n.[51G > A]; [55G > A]	Targeted trio exome sequencing (FFPE of placental tissue after TOP; 120 days after TOP)	TOP at 33 + 6 weeks	1 TOP due to fetal brain anomalies, 1 healthy daughter 1 ongoing unaffected pregnancy (CVS)

*Note:* Cases 1 and 2 share the same parents. * Variants are classified as pathogenic based on ACMG criteria (PS3, PS4, PM2, PM3).

Abbreviations: AC = amniocentesis; ACC = agenesis of corpus callosum; CVS = chorionic villus sampling; F/U = follow up; IUGR = intrauterine growth restriction; MA = maternal age; MOPD1 = Microcephalic Osteodysplastic Primordial Dwarfism type, TOP = termination of pregnancy.

For CMA testing an oligo‐array (180k Agilent, design 086332) was used. For exome sequencing (ES), samples were processed with the SureSelect Human All Exon v7 kit (Agilent). Samples for genome sequencing (GS) were processed using the TruSeq DNA PCR‐Free kit (Illumina). Sequencing was performed in paired‐end mode on a NovaSeq6000 system (Illumina) and data were analyzed using an in‐house bioinformatics pipeline (megSAP, https://github.com/imgag/megSAP). For targeted ES enrichment customized Twist Exome was used and sequenced with NovaSeq6000 (Illumina). Sanger sequencing was performed using the corresponding PCR‐amplified gene sequence and analyzed using SeqPilot‐Software (JSI medical systems). The identified gene variants were evaluated, and their pathogenicity was classified according to the American College of Medical Genetics and Genomics (ACMG) standards and guidelines. The study was approved by the Institutional Research Ethics Board (012/2025BO2).

## 3 Results

### 3.1 Prenatal Ultrasound Scans

(See Table 2) Three cases underwent first‐trimester ultrasound with detailed organ screening, which was normal (Case 1,2, and 5, Table 2). Prenatal ultrasound evaluations performed before 18 + 2 weeks were all normal. IUGR was the first anomaly detected in all five cases (at 18 + 2 weeks, 18 + 5 weeks, 20 + 1 weeks, 20 + 2 weeks and 23 + 1 weeks, respectively). All five fetuses presented with consistent prenatal sonographic anomalies including microcephaly, IUGR, ACC, intracranial cysts, lissencephaly and micrognathia. In addition to IUGR, ACC was detected in Case 2 at 20 + 1 week and myocardial echogenic focus in Case 5 at 18 + 2 weeks (Table 1). Two cases showed dilated cisterna magna (Case 2 and 5). Prenatal ultrasound findings suggestive of skeletal dysplasia, such as right hand fixated in ulnar deviation, were detected in one case (Case 3, Table 2). The detailed fetal biometric data for the five cases are summarized in Table S1.

**TABLE 2:** Summarized prenatal ultrasound findings at 20+ weeks in fetuses with *RNU4ATAC* variants.

	Case 1	Case 2	Case 3	Case 4	Case 5	Cases per feature
Brain anomalies						
Lissencephaly	+	+	−	+	+	4/5
Microencephaly	+	+	+	+	+	5/5
Hypoplastic frontal lobes	−	−	+	−	−	1/5
Intracranial cyst	+	+	+	−	+	4/5
Cerebellar vermis agenesis/hypoplasia	+	+	+	−	+	4/5
Agenesis of corpus callosum (ACC)	+	+	+	+	+	5/5
Others anomalies	−	Dilated cisterna magna	−	−	Dilated cisterna magna	2/5
Facial anomalies						
Microcephaly	+	+	+	+	+	5/5
Sloping forehead	+	−	−	−	−	1/5
Midface hypoplasia	−	−	−	−	−	0/5
Hypertelorism	+	−	−	−	−	1/5
Eyes/orbital region (specify)	+ Small	−	−	−	−	1/5
Micrognathia	+	+	+	−	+	4/5
Retrognathia	+	−	−	−	−	1/5
Absent nasal bone	+	−	−	−	−	1/5
Hand/foot anomalies						
Brachydactyly	−	−	−	−	−	0/5
Syndactyly	−	−	−	−	−	0/5
Other (specify)	−	−	Club foot (left), right hand fixated with ulnar deviation	−	−	1/5
Skeletal anomalies						
Mesomelia	−	−	−	−	−	0/5
Disproportion	−	−	−	−	−	0/5
Spine anomalies	−	−	−	−	−	0/5
Fractures	−	−	−	−	−	0/5
Other anomalies						
Cardiac anomalies	Hyperechogenic myocardium	−	−	−	−	1/5
Kidney anomalies	−	−	−	−	Mild dilated renal pelvis (4.1 mm)	1/5
Other organ anomalies	−	−	Hyperechogenic bowl	−	−	1/5

*Note:* + = feature present; − = feature not present.

In three of the five cases studied, TOP was performed prior to obtaining genetic results due to dismal clinical prognosis (Case 1, 2 and 5). One couple received the genetic results before TOP (Case 4), whereas another couple received the genetic results and decided to continue the pregnancy (Case 3).

### 3.2 Genetic Results

(See Table 1) Four fetuses were female (46,XX) and one fetus was male (46,XY). CMA was normal in all 5 cases. All fetuses carried recurrent biallelic pathogenic *RNU4ATAC* variants located in the 5′ stem loop previously associated with the clinical phenotype of MOPD1. Prenatal trio exome sequencing failed to detect the non‐coding *RNU4ATAC* variants in one case (Case 1). Targeted exome sequencing (case 3–5) or genome sequencing (Case 2) detected the variants either prenatally or after TOP. Three fetuses were homozygous for the n.51G > A variant (case 1–3), Case 4 was compound‐heterozygous for variants n.51G > A and n.30G > A and Case 5 was compound‐heterozygous for variants n.51G > A and n.55G > A. In all cases, the parents were heterozygous carriers for one of the *RNU4ATAC* variants.

### 3.3 Detailed Case Descriptions

**FIGURE 1:**
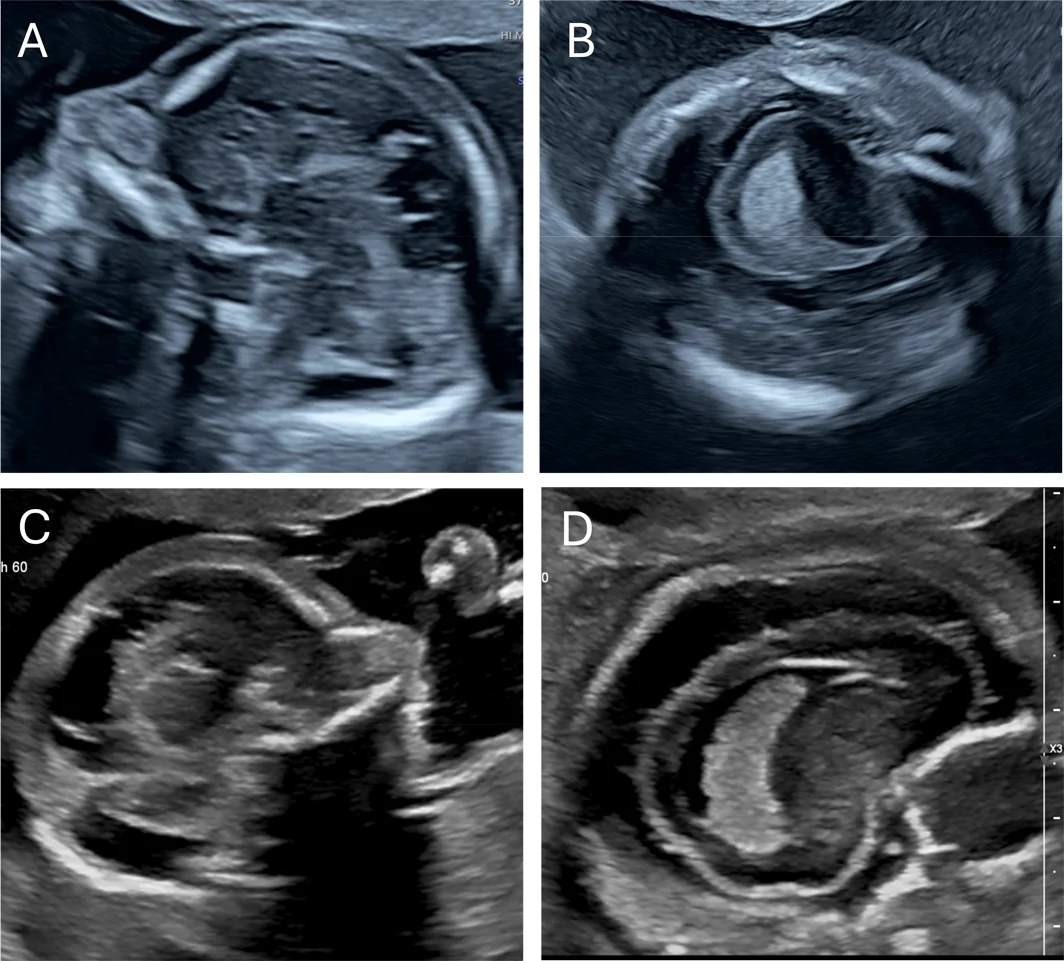
Ultrasound examinations at 20 + 2 weeks of gestation (case 1, A and B) and at 20 + 5 weeks of gestation (case 2, C and D) show lissencephaly with smooth surface of the cerebral hemispheres and wide extracerebral fluid space, sloping forehead as expression of the hypoplastic calvarium/microcephaly, intracranial cysts, and micro‐/retrognathia. Image A demonstrates an absent nasal bone.

The mother of Cases 1 and 2 had a history of two spontaneous miscarriages in the first trimester (10 and 9 weeks of gestation) and one healthy son. During the pregnancy with Case 1 (maternal age 32 yrs), prenatal ultrasound revealed IUGR, microcephaly, ACC, intracranial cysts, lissencephaly, absent nasal bone, hyperechoic myocardium, micrognathia and retrognathia (Figure 1A, B). Fetal CMA and fetal and parental karyotyping were normal. Prenatal parent‐fetus trio exome sequencing was inconclusive. TOP was performed at 22 + 0 weeks. Autopsy confirmed the ultrasound findings and demonstrated additional features such as bilateral transverse palmar creases, rocker bottom feet and nuchal translucency.

During the pregnancy with Case 2 (maternal age 35 yrs) first‐trimester screening at 11 + 5 weeks was normal. At 20 + 1 week, similar fetal malformations to those observed in Case 1 were detected (IUGR, microcephaly, lissencephaly, ACC) and midline intracranial cysts (Figure 1C, D). NIPT was within normal limits. Fetal chromosome analysis was normal. TOP was performed at 20 + 6 weeks. Parents declined autopsy. Parent‐fetus trio GS performed after TOP detected the homozygous *RNU4ATAC* n.51G > A variant in Case 2. Segregation analysis using Sanger sequencing of stored fetal DNA from Case 1 confirmed the same familial homozygous *RNU4ATAC* variant in Case 1.

The mother of Case 3 had a history of one spontaneous miscarriage in the first trimester (8 weeks of gestation) and has two unaffected children. In her second pregnancy (Case 3), prenatal ultrasound at 20 + 2 weeks showed IUGR, microcephaly, micrognathia, intracranial cysts, hypoplastic frontal lobes, cerebellar vermis hypoplasia and ACC. Furthermore, a hyperechogenic bowl, a left‐sided club foot and the right hand fixated in ulnar deviation were detected. Amniocentesis was performed at 24 + 6 weeks. Fetal karyotyping was normal and CMA was inconclusive. Targeted parent‐fetus trio ES detected the homozygous *RNU4ATAC* n.51G > A variant. A fetal MRI was performed at 29 + 3 weeks. The child was born at 39 + 1 weeks with 1425g (< 1st percentile, −5 SD), body length of 40.5 cm (< 1st percentile, −5SD) and head circumference of 26.0 cm (< 1st percentile, −8 SD). In addition to the prenatal ultrasound findings, postnatal examination revealed hypertelorism, deep set dysplastic ears, right‐sided pes calcaneus, bilateral hip dysplasia Graf type IV and arthrogryposis involving large joints as well as hands and fingers. The child suffered from severe failure to thrive and hypotonia. It was fed by a nasogastric tube and had multiple infections. At the age of 30 months, the child died of sepsis. In the third and fourth pregnancy, prenatal diagnosis was performed by CVS with targeted testing for the familial *RNU4ATAC* variants, and both fetuses were unaffected.

The mother of Case 4 has only been pregnant with Case 4. Prenatal ultrasound at 23 + 1 week showed IUGR, microcephaly, lissencephaly and ACC. NIPT was within normal limits. Fetal CMA and chromosome analysis were normal. Targeted parent‐fetus trio ES was performed and the compound‐heterozygous variants n.51G > A and n.30G > A were detected, confirming MOPD1. Genetic results were available at 25 + 3 weeks and TOP was performed at 27 weeks.

The mother of Case 5 had a previous healthy daughter and a prior TOP at 20 weeks due to fatal malformations reported as holoprosencephaly, microcephaly and micrognathia. Fetal CMA and karyotyping were normal in this pregnancy and no further testing was performed. No autopsy was performed. In a consecutive pregnancy (Case 5), prenatal ultrasound showed IUGR, microcephaly, ACC, intracranial cysts, cerebellar vermis agenesis, dilated cisterna magna and micrognathia. NIPT was within normal limits. TOP was performed at 33 + 6 weeks. Targeted parent‐fetus trio ES was performed on DNA extracted from placental tissue (FFPE) after TOP. The compound‐heterozygous *RNU4ATAC* variants n.51G > A and n.55G > A were detected. DNA for retrospective *RNU4ATAC*‐analysis was not available from the previous fetus. In a fourth pregnancy, prenatal diagnosis was performed by CVS with targeted testing for the familial *RNU4ATAC* variants, and the fetus was unaffected.

## 4 Discussion

Our study expands the existing knowledge on prenatal presentations associated with biallelic pathogenic variants in the *RNU4ATAC* gene associated with MOPD1. All fetuses exhibited characteristic and consistent prenatal anomalies, including microcephaly, IUGR, ACC, intracranial cysts, lissencephaly, and micrognathia, thus proposing that *RNU4ATAC*‐related MOPD1 might manifest distinctly during fetal development. The literature on RNU4ATAC‐opathies predominantly focuses on postnatal aspects of the disorder, with only a few reports addressing prenatal features. To our knowledge, there have been 7 fetuses with *RNU4ATAC*opathy described prenatally in the literature so far [4, 5, 6]. Six of them have been associated with MOPD1 and one fetus with Roifman syndrome. Two of these MOPD1 fetuses presented with severe microcephaly and brain anomalies such as intracranial cysts in the second trimester on prenatal ultrasound; growth retardation was not described [4]. Four fetuses were analyzed and diagnosed with MOPD1 by the fetopathology department after TOP; prenatal ultrasound information for those fetuses is not available [5]. The fetus with Roifman syndrome did not present with microcephaly but with hydrocephalus and a relatively large head, mild growth retardation and brain anomalies such as vermis hypoplasia and ACC on prenatal ultrasound [6].

In our study, first‐trimester ultrasound carried out by Maternal‐Fetal Medicine specialists was unremarkable when performed (3/5 cases). IUGR was a common first prenatal anomaly in the second trimester (18 weeks). Additionally, consistently observed features in our cohort included microcephaly and ACC, which can be detected during second‐trimester screening and correlate with the neurodevelopmental abnormalities reported in previous postnatal cases [8, 9].

Since IUGR may indicate placental insufficiency, it should be noted that microcephaly is markedly pronounced in MOPD1 cases, suggesting a non‐placental cause.

One notable finding in our cohort is the absence of pronounced prenatal skeletal dysplasia in our cohort, in contrast to the postnatal phenotype of MOPD1, in which skeletal abnormalities are hallmark features [2, 8, 9, 10]. Instead, our data highlight the prominence of IUGR, microcephaly, craniofacial features such as micrognathia and brain malformations in the prenatal setting. This phenotypic nuance expands the clinical spectrum of MOPD1 in the prenatal period and underscores the importance of considering *RNU4ATAC* variants even in the absence of classic skeletal dysplasia. This finding is consistent with reports by Wang et al., who described fetuses with RNU4ATAC‐opathies presenting with severe microcephaly and brain anomalies on prenatal ultrasound, without evidence of skeletal abnormalities [4].

Interestingly, Putoux et al. described antenatal features in four fetuses with MOPD1. The phenotyping was done after TOP in the pathology department. Here, signs of skeletal dysplasia such as brachydactyly, short limbs and ossification delay were detected in all fetuses, tapering fingers in two fetuses (twins; compound heterozygous n.40C > T and n.124G > A) and flexion contractures in the remaining two fetuses (homozygous n.51G > A; compound heterozygous n.51G > A and n.124G > A). The twin fetuses showed mild growth retardation, whereas the other two fetuses showed more pronounced growth retardation when examined by the pathologists.

The apparent discrepancies in fetal findings across studies and assessment methods may be explained by differences in sensitivity and technical limitations of prenatal ultrasound, as well as by individual variability and differences in developmental stage at the time of evaluation. Hence, it might be possible that certain skeletal dysplasia features are not prominent enough to be readily detected on ultrasound in utero.

From a technical perspective in genetic analysis, our cases highlight limitations of standard prenatal ES in detecting pathogenic variants in non‐coding RNA genes such as *RNU4ATAC*. Non‐coding genes are typically missed by standard ES, as conventional exome capture kits are primarily designed to target protein‐coding regions and therefore do not systematically cover non‐coding RNA genes. RNA splicing is a crucial step in the maturation of precursor mRNAs. Its dysregulation due to pathogenic variants in both non‐coding RNAs and protein‐coding RNAs is associated with a wide range of human diseases. Recently, genome sequencing‐based analysis of spliceosomal snRNA genes has enabled the identification of various non‐coding genes, including RNU4‐2 and RNU2‐2, as common causes of neurodevelopmental disorders [11, 12]. It can be assumed that the analysis of non‐coding genes will play a greater role in prenatal diagnostics in the future.

Existing prenatal genomic cohorts likely underrepresent fetuses with pathogenic variants in *RNU4ATAC* and other non‐coding genes. This is largely because prenatal ES, rather than prenatal GS, remains the standard diagnostic approach, and targeted testing of non‐coding regions is not routinely pursued in cases with non‐specific fetal phenotypes. The fact that one of our cases had negative prenatal trio ES results but was subsequently diagnosed by GS highlights the need to include *RNU4ATAC* in targeted panels or to consider prenatal GS, especially when multiple fetal anomalies raise suspicion but WES remains inconclusive. This approach should be considered particularly in cases of recurrent pregnancies with consistent prenatal findings. Although the financial cost and technical burden of GS are expected to decline, the analysis of larger datasets may still pose challenges in the prenatal setting, where time constraints and limited resources are critical factors.

There were several limitations to this study. It is a retrospective analysis and the cases were from different institutions and medical systems; therefore, the methods used to establish a genetic diagnosis are different. All fetuses were examined by Maternal‐Fetal‐Medicine specialists but the experience and ultrasound systems used may have varied. Our series consists of five fetuses, which is a decent amount given the rarity of the disease. However, even though all *RNU4ATAC*‐variants detected are associated with MOPD1, not all fetuses carry the same *RNU4ATAC* variants, which could partly explain differences in prenatal phenotype or variant‐specific expressivity.

In summary, our results show that *RNU4ATAC* variants associated with MOPD1 can cause a recognizable prenatal constellation of IUGR, microcephaly and structural brain malformations, possibly with subtle skeletal involvement, which should prompt inclusion of this gene in prenatal diagnostic panels or offering GS in the prenatal setting. Increased awareness of this potential diagnostic gap in standard ES may improve diagnostic yield and support earlier detection, while also supporting prenatal counseling and reproductive decision making. Ultimately, shortening the time to diagnosis by integrating technologies that capture non‐coding variants might improve patient care in the prenatal setting.

## Funding

The authors have nothing to report.

## Ethics Statement

The study was approved by the ethics committee of the University Hospital of Tuebingen (012/2025BO2) on May 2nd 2024.

## Consent

Written informed consent was obtained from the parents for publication of the cases.

## Conflicts of Interest

The authors declare no conflicts of interest.

## Supporting information


**Table S1:** Fetal biometry of the five cases, expressed as percentiles and z‐scores of the NICHD Fetal Growth Studies non‐Hispanic white standard.

## Data Availability

The data that support the findings of this study are available on request from the corresponding author. The data are not publicly available due to privacy or ethical restrictions.
