# Supplementary material for: Prenatal Phenotypic Features of Five Fetal Cases With RNU4ATAC‐Associated Microcephalic Osteodysplastic Primordial Dwarfism Type I
Source: Prenat Diagn. 2026 Aug 14;46(10):1648–54. doi: 10.1002/pd.70240 (PMC13571799; doi:10.1002/pd.70240)
Supplement: Supplementary file 1 [file PD-46-1648-s001.docx]

Table S1 I Fetal biometry of the five cases, expressed as percentiles and z-scores of the NICHD Fetal Growth Studies non-Hispanic white standard.

| **Case** | **GA** | **BPD (mm)** | | | **HC (mm)** | | | **AC (mm)** | | | **FL (mm)** | | | **EFW (g)** | | |
| --- | --- | --- | --- | --- | --- | --- | --- | --- | --- | --- | --- | --- | --- | --- | --- | --- |
|  |  | **Value** | **Centile** | ***z*** | **Value** | **Centile** | ***z*** | **Value** | **Centile** | ***z*** | **Value** | **Centile** | ***z*** | **Value** | **Centile** | ***z*** |
| **Case 1** | 10+5 | 15.4 | 76 | +0.7 | 35.7 CRLᵃ | 28 | −0.6 | – | – | – | 3.6 | 53 | +0.1 | – | – | – |
|  | 18+5 | 33.8 | < 1 | −3.8 | 127.8 | < 1 | −4.3 | 109.0 | < 1 | −3.5 | 21.0 | < 1 | −2.7 | 158 | < 1 | −4.1 |
|  | 20+2 | 34.8 | < 1 | −4.9 | 129.2 | < 1 | −6.4 | 125.9 | < 1 | −3.4 | 26.3 | < 1 | −2.4 | 211 | < 1 | −4.4 |
|  |  |  |  |  |  |  |  |  |  |  |  |  |  |  |  |  |
| **Case 2** | 11+5 | 16.7 | 14 | −1.1 | 50.1 CRL ᵃ | 39 | −0.3 | 51.9 | 40 | −0.2 | 6.1 | 68 | +0.5 | – | – | – |
|  | 20+1 | 37.4 | < 1 | −3.7 | 143.4 | < 1 | −4.2 | 136.2 | 2 | −2.0 | 29.7 | 19 | −0.9 | 259 | < 1 | −2.5 |
|  | 20+5 | 38.7 | < 1 | −3.9 | 150.7 | < 1 | −4.1 | 142.7 | 2 | −2.0 | 32.0 | 26 | −0.6 | 294 | < 1 | −2.4 |
|  |  |  |  |  |  |  |  |  |  |  |  |  |  |  |  |  |
| **Case 3** | 20+2 | 40.0 | < 1 | −2.8 | 150.0 | < 1 | −3.5 | 125.0 | < 1 | −3.5 | 33.0 | 56 | +0.2 | 264 | < 1 | −2.6 |
|  |  |  |  |  |  |  |  |  |  |  |  |  |  |  |  |  |
| **Case 4** | 23+1 | 45.7 | < 1 | −3.7 | 175.8 | < 1 | −4.4 | 168.4 | 2 | −2.1 | 38.8 | 28 | −0.6 | 448 | < 1 | −2.4 |
|  |  |  |  |  |  |  |  |  |  |  |  |  |  |  |  |  |
| **Case 5** | 14+2 | 25.8 | 16 | −1.0 | 95.3 | 11 | −1.2 | 77.2 | 18 | −0.9 | 14.3 | 69 | +0.5 | 90 | 32 | −0.5 |
|  | 18+2 | 36.5 | 3 | −1.9 | 136.6 | 1 | −2.3 | 115.1 | 2 | −2.1 | 27.1 | 57 | +0.2 | 201 | 7 | −1.5 |
|  | 22+2 | 45.1 | < 1 | −3.1 | 172.2 | < 1 | −3.6 | 158.8 | 2 | −2.1 | 37.6 | 42 | −0.2 | 403 | 2 | −2.1 |
|  | 26+4 | 56.1 | < 1 | −3.5 | 214.0 | < 1 | −4.0 | 193.4 | < 1 | −3.1 | 47.2 | 28 | −0.6 | 708 | < 1 | −3.0 |
|  | 28+6 | 62.9 | < 1 | −3.1 | 230.6 | < 1 | −4.5 | 211.9 | < 1 | −3.4 | 53.4 | 44 | −0.1 | 946 | < 1 | −3.1 |
|  | 31+2 | 66.5 | < 1 | −3.8 | 244.1 | < 1 | −5.1 | 226.9 | < 1 | −3.9 | 57.0 | 27 | −0.6 | 1148 | < 1 | −3.7 |
|  | 32+6 | 70.4 | < 1 | −3.5 | 255.8 | < 1 | −4.8 | 248.6 | < 1 | −3.3 | 59.1 | 18 | −0.9 | 1391 | < 1 | −3.5 |

GA = gestational age (weeks+days); BPD = biparietal diameter; HC = head circumference; AC = abdominal circumference; FL = femur length; EFW= estimated fetal weight; CRL = crown–rump length; – = not available.

Since the fetuses were examined using different ultrasound systems with varying growth curves, all ultrasound measurements were reported as Z-scores and percentiles relative to a single reference to ensure comparability.

Reference standard: NICHD Fetal Growth Studies race/ethnicity-specific standards, non-Hispanic white cohort (Buck Louis GM, Grewal J, Albert PS, et al. Racial/ethnic standards for fetal growth: the NICHD Fetal Growth Studies. Am J Obstet Gynecol 2015;213:449.e1–449.e41; Table 2), covering gestational weeks 10–40.

Percentiles and z-scores were obtained by linear interpolation of the published percentile curves (3rd–97th) to the exact gestational age, followed by interpolation between the two adjacent curves on the probit scale. Measurements beyond the published range were extrapolated linearly on the probit scale; because the reference cohort comprises 614 women, percentiles beyond the 1st are not supported by the source data and are therefore reported as "< 1", with the z-score given as the continuous measure of deviation.

EFW was calculated from HC, AC and FL using the Hadlock formula applied in the NICHD study and referenced to the NICHD non-Hispanic white EFW standard.

ᵃ CRL measurement; the NICHD standards contain no CRL reference, and the corresponding centile and z-score were derived from the Robinson & Fleming curve (Robinson HP, Fleming JEE. A critical evaluation of sonar crown–rump length measurements. Br J Obstet Gynaecol 1975;82:702–710).
